# Metastatic rectal adenocarcinoma within haemorrhoids: a case report

**DOI:** 10.1186/1752-1947-2-128

**Published:** 2008-04-28

**Authors:** Dorothy M Gujral, Sanjeev Bhattacharyya, Peter Hargreaves, Gary W Middleton

**Affiliations:** 1 St Lukes Cancer Centre, Royal Surrey County Hospital, Egerton Road, Guildford, Surrey GU2 7XX, UK

## Abstract

**Introduction:**

Metastatic tumour involvement of the anal canal is rare. Routine pathological evaluation of haemorrhoidectomy specimens has been suggested to be unhelpful and expensive. Selective rather than routine pathological evaluation of haemorrhoidectomy specimens has been recommended.

**Case presentation:**

We report the case of a 69-year-old woman with metastatic colorectal carcinoma who presented with metastatic carcinoma within thrombosed haemorrhoids.

**Conclusion:**

We suggest that in patients with colorectal cancer, careful examination of haemorrhoids on colonoscopy as well as histological examination of suspected haemorrhoidal tissue after surgical resection be performed to evaluate for metastasis.

## Introduction

Metastatic tumour involvement of the anal canal is rare. There have been around 200 cases of metastatic anorectal melanoma, with the first case described in 1857 by Moore [1]. Other non-colorectal tumours in the anal canal are very rare. Metastatic tumour involvement of the anal canal from squamous cell carcinoma, anaplastic carcinoma of the lung and breast cancer has previously been reported [2-4]. Metastatic cancer to haemorrhoidal tissue is even rarer [5,6].

Lemarchand et al. [7] performed a retrospective analysis of haemorrhoidectomy specimens obtained in a coloproctology unit between 1 January 1985 and 31 December 2001. Fifty-six histological abnormalities (0.69%) were detected among 8153 haemorrhoidectomy specimens considered normal at gross macroscopic examination. The authors concluded that routine pathological evaluation of haemorrhoidectomy specimens was not useful and was expensive. The authors also concluded that selection for gross and microscopic evaluation of suspicious areas at the preoperative examination should be continued.

A study by Cataldo et al. [8] looked at haemorrhoidectomy specimens taken from 21,257 patients over a 20-year period. They noted only one instance of unsuspected carcinoma of the anus diagnosed solely by microscopic analysis of a specimen that was taken at haemorrhoidectomy, and the authors recommended selective rather than routine pathological evaluation of haemorrhoidectomy specimens. It would be reasonable to assume that patients with known carcinoma would be at higher risk of developing haemorrhoidal metastases.

## Case presentation

A 69-year-old woman presented in February 2003 with a 6-week history of progressive change in bowel habit. She had noticed fresh blood mixed with her stools, a 1-stone weight loss, and pelvic pain.

Blood tests showed an elevated carcino-embryonic antigen (CEA) of 560 ng/ml and colonoscopy revealed a fixed, circumferential rectal carcinoma at 6 cm. No other synchronous tumours were noted to the level of the caecum. Computed tomography scan showed bilateral pulmonary metastases, multiple liver metastases, and a large mass in the recto-sigmoid region consistent with known carcinoma. Magnetic resonance imaging of the pelvis confirmed a large infiltrative tumour seen in the upper rectum with ill-defined margins. Rectal biopsy confirmed a moderate to poorly differentiated adenocarcinoma of large bowel type.

The patient was commenced on palliative chemotherapy with irinotecan and infusional 5-FU. After 14 cycles, the patient unfortunately developed progressive disease with new pulmonary metastases and an increase in the size of her liver metastases as well as an increase in her CEA from 113 ng/ml to 710 ng/ml. Consequently, in January 2004, treatment was changed to oxaliplatin and infusional 5-FU chemotherapy.

**Figure 1:**
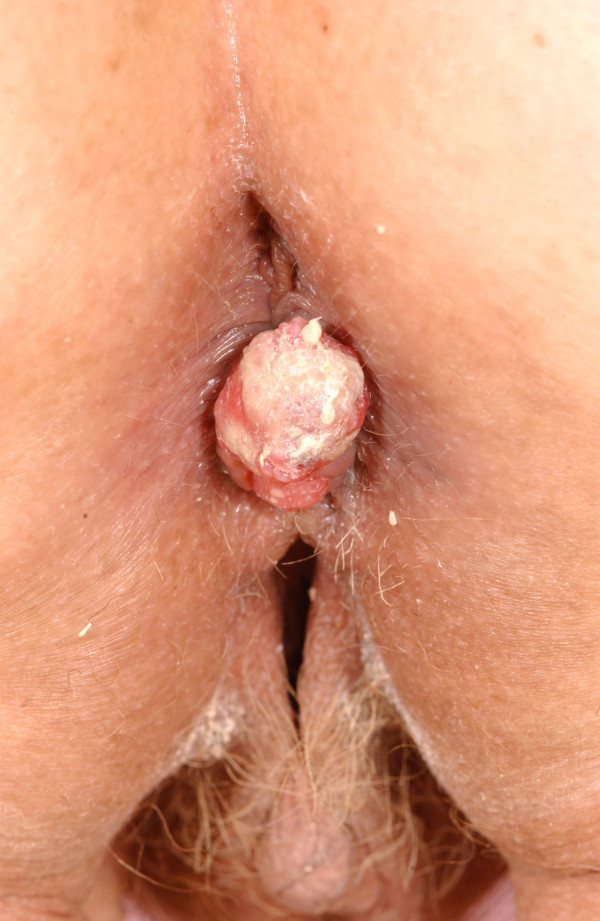
Pedunculated, hard, ulcerating haemorrhoids with tumour deposits.

**Figure 2:**
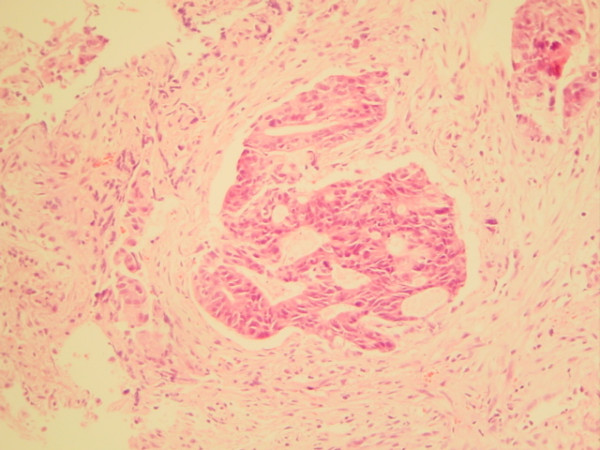
Original biopsy of rectal tumour demonstrating adenocarcinoma.

**Figure 3:**
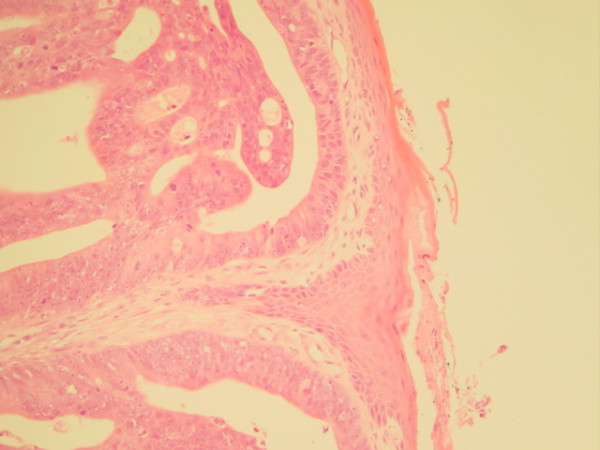
Biopsy of haemorrhoidectomy specimen with adenocarcinoma within anal canal squamous tissue.

After three cycles, the patient presented complaining of prolapsed 'piles' and on examination was found to have pedunculated, hard and ulcerating haemorrhoids. Tumour deposits were noted at the 6 and 12 o'clock positions (Figure 1). These were subsequently excised and microscopy revealed deposits of moderate to poorly differentiated adenocarcinoma of large bowel type similar to the original rectal biopsy (Figures 2 and 3).

## Conclusion

This case is, to the best of our knowledge, the first case that demonstrates bowel adenocarcinoma deposits within haemorrhoids. We suggest that in patients with a history of colorectal cancer, careful examination of haemorrhoids during colonoscopy, as well as histological examination of suspected haemorrhoidal tissue after surgical resection, be performed to evaluate for the possible presence of metastasis.

## Competing interests

The authors declare that they have no competing interests.

## Authors' contributions

DMG, PH and GWM were all involved in the clinical care of the patient. DMG, SB and PH conceived, researched, wrote the paper and revised the final manuscript. All authors read and approved the final manuscript.

## Consent

Written consent was obtained from the patient's next-of-kin for publication of this case report and accompanying images. A copy of the written consent is available for review by the Editor-in-Chief of this journal.

## References

[B1] Moore Recurrent melanomas of the rectum, after previous removal from the verge of the anus, in a man age sixty-five Lancet 1857 1 290 10.1016/S0140-6736(02)20230-5

[B2] Rueben J Ger R Squamous cell carcinoma of the anal canal: a metastatic lesion Dis Colon Rectum 1968 11 213 219 5666898 10.1007/BF02617298

[B3] Kanhouwa S Burns W Matthews M Chisholm R Anaplastic carcinoma of the lung with metastasis to the anus: report of a case Dis Colon Rectum 1975 18 42 48 10.1007/BF02587239 1126255

[B4] Dawson PM Hershman MJ Wood CB Metastatic carcinoma of the breast in the anal canal Postgrad Med J 1985 61 1081 4095054 10.1136/pgmj.61.722.1081 PMC2418533

[B5] Sawh RN Borkowski J Broaddus R Metastatic renal cell carcinoma presenting as a haemorrhoid Arch Pathol Lab Med 2002 126 856 858 12088459 10.5858/2002-126-0856-MRCCPA

[B6] Timaran CH Sangwan YP Solla JA Adenocarcinoma in a haemorrhoidectomy specimen: case report and review of the literature Am Surg 2000 66 789 792 10966042

[B7] Lemarchand N Tanne F Aubert M Benfredj P Denis J Dubois-Amous N Fellous K Ganansia R Senejoux A Soudan D Puy-Montbrun T Is routine pathologic evaluation of haemorrhoidectomy specimens necessary? Gastroenterol Clin Biol 2005 29 213 217 15646532 10.1016/s0399-8320(04)95044-0

[B8] Cataldo PA Mackiegan JM The necessity of routine pathologic evaluation of haemorrhoidectomy specimens Surg Gynecol Obstet 1992 174 302 304 1553609

